# WIC's promotion of infant formula in the United States

**DOI:** 10.1186/1746-4358-1-8

**Published:** 2006-04-20

**Authors:** George Kent

**Affiliations:** 1 Department of Political Science, University of Hawai'i, Honolulu, Hawai'i 96822, USA

## Abstract

**Background:**

The United States' Special Supplemental Nutrition Program for Women, Infants and Children (WIC) distributes about half the infant formula used in the United States at no cost to the families. This is a matter of concern because it is known that feeding with infant formula results in worse health outcomes for infants than breastfeeding.

**Discussion:**

The evidence that is available indicates that the WIC program has the effect of promoting the use of infant formula, thus placing infants at higher risk. Moreover, the program violates the widely accepted principles that have been set out in the International Code of Marketing of Breast-milk Substitutes and in the human right to adequate food.

**Summary:**

There is no good reason for an agency of government to distribute large quantities of free infant formula. It is recommended that the large-scale distribution of free infant formula by the WIC program should be phased out.

## Background

### Anomalies

More than half the infant formula used in the United States is provided to mothers at no cost through the federal government's Special Supplemental Nutrition Program for Women, Infants, and Children, commonly known as WIC. In 2000, Cynthia Tuttle asked:

Why does the WIC program continue to be the largest promoter of formula feeding to low-income women in the United States, in terms of the program's provision of formula products [[1], p100]?

The challenge provoked fascinating discussion in the journal, but no clear answer. The question still stands. Several related questions can be raised as well.

WIC is intended to serve low-income women, infants, and children who are at nutritional risk. WIC reaches almost half of all US infants. Are that many infants in the US low income or at nutritional risk?

WIC provides more than half of the formula that is used in the US, but serves less than half the infants. This means that infants who are WIC clients are more likely to get formula than infants who are not in the WIC program. Why?

Infant formula is costly. Why is it that low-income people are more likely to use infant formula than high-income people?

Does WIC's distribution of infant formula encourage low-income people to use it? Should low-income people be encouraged to use it?

US policy supposedly is to encourage free market competition. Why is it that just three manufacturers dominate the infant formula market in the US? Why does the US government support this triopoly? Why does the government support this triopoly despite the fact that it increases the cost of formula to those who are not WIC clients?

WIC operations are funded in part by large rebates to WIC from the formula companies. These rebates are not treated as charitable contributions. How is giving such large rebates economically sensible for the formula companies?

Internationally accepted standards say that advertising for infant formula should be limited. How is it that US government publications themselves carry pictures that highlight specific brands of infant formula?

Several branches of the US government promote breastfeeding. WIC itself has a program for promoting breastfeeding. Why then does WIC distribute formula free in massive quantities?

Why should WIC provide any formula at all to its clients when it is known to be less healthful for infants than breastfeeding?

### The inferiority of infant formula

Worldwide, more than ten million children die before their fifth birthdays each year [2]. About half of these deaths are associated with nutrition problems [3]. Many studies demonstrate that there is a close linkage between infant feeding patterns and mortality. For example, in one study in Brazil, "infants who received powdered milk or cow's milk, in addition to breast milk, were at 4.2 times ... the risk of death from diarrhea compared with infants who did not receive artificial milk, while the risk for infants who did not receive any breast milk was 14.2 times higher ... Similar results were obtained when infants who died from diarrhea were compared with infants who died from diseases that were presumed to be due to noninfectious causes" [[4], p1032].

The use of breast-milk substitutes such as infant formula has impacts on the infant, the family, and the broader society on many dimensions. With regard to the health impacts on infants, the use of infant formula tends to increase the illness and deaths associated with a wide range of diseases [5-10]. It has been estimated that "If every baby was exclusively breastfed from birth for six months, an estimated 1.3 million lives could be saved each year" [[11], p1]. Relatively few of those excess deaths occur in the richer countries.

The infant mortality associated with not breastfeeding is relatively small in richer countries, but it is not negligible. Recent studies indicate that formula feeding in the US causes substantial numbers of excess infant deaths. The risk of post-neonatal (29–365 days of age) mortality is about 27% higher among infants who are never breastfed compared to infants who are ever breastfed. On this basis, about 720 infant deaths in the US would be averted each year if all infants were breastfed [12].

This refers to postnatal infant deaths, meaning deaths that occur beyond 28 days after birth, but before the first birthday. This estimate of the impact of not breastfeeding may be understated for several reasons. One consideration is that Chen and Rogan's study excluded neonatal deaths (0–28 days). Second, "because exposure to breastfeeding was categorized based on the infant ever being breastfed, the estimate is based on a mixture of breastfeeding exposure levels, including many who were breastfed for a very brief period." Thus, "the estimate of 720 lives saved is likely to be an underestimate compared to the additional effect of continued breastfeeding" [[13], p357]. Third, if deaths beyond the first year were included, the estimate for the number of deaths associated with not breastfeeding would be higher.

Apart from the higher mortality, there are many illnesses that occur at higher rates as a result of not breastfeeding. In rich countries as well as poor, the consequences of not breastfeeding are more likely to show up as illness rather than death.

There is far less morbidity (illness) and mortality (death) resulting from using formula in richer countries than in poorer countries. Nevertheless, just about every study that compares the health consequences of breastfeeding with the health consequences of using formula in a population shows that formula leads to worse health consequences for infants.

The US government's Office on Women's Health identifies the major benefits of breastfeeding for the infant in terms of nutrition and growth benefits and also enhanced immune systems and resistance to infection, and also describes the health benefits for the mother [14]. The American Dietetic Association provides similar information on the health benefits of breastfeeding to both infant and mother [15].

The choice of feeding methods also has significant economic consequences, at the national level as well as at the family level. Breastfeeding yields savings not only because of the elimination of the cost of infant formula but also because of averted health care costs. A US government study estimates that in the US, "A minimum of $3.6 billion would be saved if breastfeeding were increased from current levels ... to those recommended by the U.S. Surgeon General ..., This figure is likely an underestimation of the averted health care costs because it represents cost savings from the treatment of only three childhood illnesses ..." [[16], p1]. Moreover, it does not count the savings of the cost of formula. The savings would be even greater if breastfeeding in the US were to follow the recommendations of the World Health Organization. WHO recommends that all infants should be exclusively breastfed for six months, and breastfeeding should be continued, with appropriate complementary feeding, for up to two years and beyond [17].

There are few conditions under which infants should not be breastfed by their biological mothers. According to a study commissioned by the US government, there are no nutritional contraindications to breastfeeding infants except for infants with special health needs such as galactosemia or phenylketonuria [[5], p6].

Also, breastfeeding by the mother is not recommended when the mother has certain infectious diseases, has taken certain pharmaceuticals or street drugs, or has certain environmental contaminants in her breast milk. Nevertheless, "Contraindications are rare in the United States" and "Breast milk should not be withheld from any infant unless absolutely necessary" [5]. The American Academy of Pediatrics recommends, "Pediatricians and other health care professionals should recommend human milk for all infants in whom breastfeeding is not specifically contraindicated ..." [[18], p498].

Under some conditions, such as prematurity, infants may not be able to breast *feed*, but nevertheless would benefit from breast *milk*, whether from their own mothers or from donors.

It seems reasonable to estimate that no more than 5% of the infants in the US need to use breast-milk substitutes for health reasons.

## Discussion

### The WIC program

The WIC program, launched in 1974, is administered by the Food and Nutrition Service (FNS) of the US Department of Agriculture (USDA). It was authorized by the Child Nutrition Act of 1966, as amended, Section 17, 42 USC. 1786. The WIC program "serves to safeguard the health of low-income women, infants, & children up to age 5 who are at nutritional risk by providing nutritious foods to supplement diets, information on healthy eating, and referrals to health care" [[19], p1].

Pregnant, breastfeeding and postpartum women, infants, and children up to 5 years of age are eligible if: 1) they are individually determined by a competent professional to be in need of the special supplemental foods supplied by the program because of nutritional risk; and 2) meet an income standard, or receive or have certain family members that receive benefits under the Food Stamp, Medicaid or Temporary Assistance for Needy Families Program.

In fiscal year 2004, the WIC program had 7,904,000 participants. Program costs were US$3,561,200,000 for food, and US$1,276,200,000 for nutrition services and administrative costs, for a total of US$4,890,200,000. The average monthly food cost per person was US$37.54 [20]. Rebates provided about US$1.5 billion additional for nutrition services and administrative costs. Most rebates are for formula purchases, but some also come from purchases of other products such as juices and infant cereal. WIC's annual budget is now well over US$5 billion a year, more than twice the budget of the United Nations Children's Fund.

WIC has the following requirements for its infant formula:

• Complies with the definition in section 201(z) of the Federal Food, Drug and Cosmetic Act (21USC. 321(z)) and meets the requirements under section 412 of the Federal Food, Drug Act (21 USC. 350a) and regulations at 21 CFR parts 106 and 107

• Nutritionally complete infant formula not requiring the addition of any ingredients other that water prior to being served in a liquid state

• Iron-fortified, containing at least 10 milligrams of iron per liter of formula at standard dilution

• Supplies 67 calories per 100 milliliters of formula at standard dilution (i.e., 20 calories per fluid ounce of prepared formula) [[21], p1].

Most infant formulas approved for the general market in the US are acceptable for use in the WIC program.

WIC says it has assessed the impacts of formula feeding on "nutrition status." For example, a 1999 study found no problems in "achieving recommended nutrient intakes among formula-fed WIC infants 2–3 months old (all groups) for energy and WIC-target nutrients (protein, iron, calcium, vitamin A, and vitamin C)" [[22], p4]. Similar conclusions were reached for infants 4–11 months old and for children 1–3 years old. Overall, the study concluded, "There were no nutrient shortfalls associated with WIC infants up to the age of 11 months" [[22], p14].

However, it should be recognized that the intakes were judged to be adequate only with regard to limited lists of specific nutrients. No notice was taken of other elements, such as immune factors, that are not available from infant formula. One must question whether nutrient intakes really are a suitable measure of nutrition status. Adults would not be able to persuade their doctors to assess their nutrition status by sending them lists of what they had eaten over the past month.

No output measures were made in terms of subsequent health consequences. No studies were done to compare the health outcomes of formula-fed infants and breastfed infants. Instead of using the health outcomes for breastfed infants as the "gold standard" against which feeding methods should be assessed, the study only measured intake against a short list of nutrients.

### WIC formula economics

In 2004 the US Department of Agriculture, which administers the WIC program, published an article on "Sharing the Economic Burden: Who Pays for WIC's Infant Formula?" It explained that WIC clients get the formula free. WIC, and thus the US Treasury, pays for it. However, WIC negotiates contracts with the formula companies under which WIC gets rebates from the manufacturers. The rebates are quite large:

Rebates per can of formula also vary across States and ranged from 85 to 98 percent of the manufacturer's wholesale price in fiscal 2000. As a result, the highest net price a manufacturer received for WIC-provided infant formula was only 15 percent of the wholesale price. Net prices in September 2000 ranged from 76.5 cents (per can of milk-based liquid concentrate) in Florida to 44.7 cents in Nebraska and South Dakota. For the US as a whole, net prices averaged 18 cents per can in fiscal 2000 [[23], p33].

As a result, "With rebates from the formula manufacturers, the cost of the formula to taxpayers is a small fraction of its wholesale price" [[23], p36]. This sounds good. But then what is the answer to the question posed in the study's title: Who pays for WIC's formula? If it is not the clients who are paying, and the taxpayers cover only a small portion of the cost, who is paying? And why?

It appears that the manufacturers are paying most of the actual cost of the product itself, which is not very much. US taxpayers are paying to distribute it, which is a substantial cost. Four factors seem to explain this curious situation: low product cost, inflated retail prices, brand loyalty, and expanded reach.

#### Low product cost

It may be that there is really is little or no loss to the manufacturers as a result of the rebates. The rebates are a large percentage of the wholesale price (85 to 98 %), but that price may be much higher than the actual cost of manufacturing the product. This view is supported by a 1998 report from the US government's General Accounting Office, which concluded, "wholesale prices of infant formula appear to be high in relation to the costs of production indicating the likelihood of high profit margin" [[24], p11]. In 1994, retail prices were estimated to be as much as five times the cost of manufacture [[25], p115]. Apparently the companies can give large rebates because their costs are much lower than the wholesale prices.

#### Inflated retail prices

The retail price of formula is high. Significantly, the retail price is higher where WIC is most active. Grocers and other merchants know that WIC will cover the retail price of formula sold through WIC vouchers, so they are motivated to push the price up. The pattern is well documented [23]. This also allows the wholesale price to creep up. Even if these price increments were relatively modest, added up across the country, they would produce a significant increase in cash flow to the manufacturers over what they could have obtained in a genuinely competitive market.

WIC's involvement produces upward pressure on retail prices. This does not affect WIC clients immediately and directly, but it does mean the price is pressed upward for those who are not WIC clients. There is in a way a cross subsidy, with non-WIC clients helping to fund formula supplies for WIC clients.

Families get free formula from WIC for only a limited time. They must face the inflated retail prices when they leave the WIC program.

The inflation of retail prices due to WIC involvement is demonstrated with great clarity in the WIC-only stores. These are "retail stores that predominantly serve WIC participants and in which the vast majority of, if not all, store revenue comes from the redemption of WIC vouchers for WIC food items." In fiscal year 2002 they accounted for 9 % of WIC sales nationwide. Market forces and competitive pricing help to limit prices in ordinary stores serving the general public, but ...

WIC-only stores, on the other hand, have no need to attract non-WIC customers and, as a result, have no incentive to set prices that are determined by market forces. Because the same market forces that have long contained costs in the WIC program for price-competitive stores do not apply to WIC-only stores, the WIC program spends considerably more for the same food items when WIC vouchers are redeemed at WIC-only stores than if those vouchers are redeemed at the average prices charged by competitive grocery stores [[26], p54–55].

In California, where over 600 WIC-only stores operate, it has been estimated that WIC food costs were about US$33 million higher than they would have been if the vouchers had been redeemed at regular grocery stores [[26], p55]. Thus, WIC-only stores are free to inflate their prices because their clients do not have to pay them, and they are guaranteed reimbursement through WIC. Most WIC food products do not produce rebates from their manufacturers to WIC, so it is US taxpayers who cover these inflated costs.

#### Brand loyalty

Why are the formula manufacturers willing to give such large rebates to the WIC program? Perhaps the history of handing out free supplies is relevant:

During the seventies, one company paid a million dollars to the City of New York for the privilege of donating free formula to all of the City hospitals. In 1989, Abbott Laboratories and Bristol Myers got into a bidding war in Canada over the exclusive right to supply free formula to Canada's largest maternity hospital [[27], p79].

Once a woman starts feeding her infant with formula, she is likely to become dependent on it, for the current infant, and possibly for future infants as well. Moreover, the manufacturer's hope is that the consumer will stay with the same brand, not only for formula but also for follow-on foods. The rebates may mean the companies lose money on their products during the period that the women are WIC clients, but they are likely to more than recover that loss if the women remain loyal to the brand after leaving WIC.

Participation in the WIC program drops off rapidly after the first year [[28], p132]. If the client is served by WIC for, say, one year, and remains a loyal customer for formula for several years after that, the investment by the manufacturer might be very worthwhile.

It may be possible for the companies to give large rebates because their costs really are much lower than the wholesale prices. Even if the rebates mean the companies suffer some loss because the net payment they receive for each can is smaller than its cost, the companies might view that loss as a cost of doing business, comparable to the cost of giving out free supplies.

#### Expanded reach

Historically, infant formula has been produced and marketed by pharmaceutical companies. Their approach to marketing was based on their well-established routines of ''medical detailing,'' the practice of contacting hospitals and medical practitioners directly, providing them with free or discounted products, and encouraging health workers to recommend their brands [[29], p8]. However, this method of marketing is quite expensive. The manufacturers of infant formula saw that a government nutrition assistance program could fulfill the same functions at much lower cost. Thus the formula manufacturers were among the strong advocates for creation of the WIC program in the 1970s. WIC now handles the logistics of distribution at little cost to the companies. As Cynthia Tuttle put it, ''The establishment of the WIC program provided formula manufacturers with a new, very direct avenue of marketing to one of their target audiences, and they were quick to take advantage of this opportunity'' [[1], p100].

In the early 1980s, many WIC offices purchased infant formula at full retail prices. However, as formula prices rose more rapidly than the prices of other foods, and formula accounted for nearly 40 % of total WIC food costs, WIC began to explore ways to limit its formula costs. In 1988 a law was passed requiring all State WIC agencies to explore cost containment procedures. Competitive bidding systems produced great savings, apparently because of the lack of strong price competition in the infant formula industry [[29], p32–33].

The generous rebates are used by WIC to expand its reach, so that it can service more clients. The effect has been dramatic: From 1982 through 1996, the percentage of infants in WIC grew from 18 % of infants born in the United States to 46 %. ''By 1997, the rebates totaled US$1.3 billion, adding 1.9 million participants to WIC, roughly one quarter of the program's entire caseload and one third of its appropriated funding'' [[24], p55]. Under the rules, the additional funds could not be used to adjust benefits or services, but had to be used to expand participation. From the manufacturers' perspective, WIC has become an effective alternative to medical detailing. The expanded reach has helped to get more infants started on formula.

The important issue here may not be the competition among the manufacturers for market share, but the fact that they collectively tend to displace the share going to mothers' milk [[25], p116]. The WIC program helps to displace that fourth option.

### Triopoly

In the year 2000, just three companies accounted for 99 % of the infant formula market in the US: Mead Johnson–52 %; Ross–35 %, and Carnation–12 % [[23], p33–34]. Each of them is a subsidiary of a larger company:

• Mead Johnson, maker of Enfamil and Gerber infant formula, is part of Bristol-Myers Squibb [30].

• Ross Laboratories, maker of Similac infant formula, is a division of Abbott Laboratories [31].

• Carnation, maker of Good Start infant formula, has been a subsidiary of Nestlé (based in Switzerland) since Nestlé purchased it in 1988 [32].

Wyeth was in the WIC infant formula market up to 1996, but dropped out after that. Only Mead Johnson, Ross, and Carnation remain. There have been some smaller companies in the business, such as Loma Linda and Rimaco, but they have left the market [[29], p33]. With the apparently high spread between manufacturing costs and wholesale prices, manufacturing infant formula must be a highly profitable business. Why aren't there more than three companies winning WIC contracts?

The government has been concerned about possible monopolistic practices in the industry that result from having only a small number of participants. In May 1990, the Senate Subcommittee on Antitrust, Monopolies, and Business Rights held a hearing on the pricing behavior of infant formula companies. The Federal Trade Commission also investigated potential anti-competitive practices in the infant formula industry. Charges of bid-rigging were brought against the three largest manufacturers [[29], p33]. While these cases have been resolved, the potential for collusion remains.

### Does WIC encourage formula use?

WIC has a vigorous and effective program for promoting breastfeeding, resulting in steadily increasing breastfeeding rates among clients. However, a USDA report acknowledges:

Although breastfeeding rates are increasing among women participating in WIC–both while in the hospital immediately after giving birth, and 6 months after giving birth–the rates continue to be lower than those of non-WIC women. Although some have questioned whether WIC provides a disincentive to breastfeeding by supplying free infant formula, the women most likely to participate in WIC, including mothers who are poor and have low education levels, are less likely to breastfeed their children in general [[23], p36].

The breastfeeding promotion program has limited impact because of its limited scale. In 2005, ''only $34 million (or 0.6% of the total WIC budget excluding rebates) was set aside for specific incentives designed to increase breastfeeding among WIC participants'' [[33], p1137]. One expert said of WIC's breastfeeding promotion program, ''the budget is small and the effort is feeble'' [[34], p1433].

Data from Ross Laboratories show that women in WIC are more likely to choose formula than comparable women who are not in WIC:

From 1978 through 2003, rates for the initiation of breastfeeding among WIC participants lagged behind those of non-WIC mothers by an average of 23.6 +/- 4.4 percentage points. At 6 months of age, the gap between WIC participants and non-WIC mothers ... steadily increased from 1978 through 2003 ... mothers who were not enrolled in the WIC program were more than twice as likely to breastfeed at 6 months of age than mothers who participated in the WIC program [[33], p1136].

It is necessary to use data from a formula manufacturer because the data published by US government agencies do not separate the information for WIC clients from information for those who do not participate in the WIC program.

Breastfeeding rates at six months have increased steadily from 1990 to 2002 for WIC participants, but they have also increased for non-WIC participants [[33], p1145]. Thus it is difficult to know the extent to which the upward trend might have been due to forces in the general population, rather than WIC's breastfeeding promotion efforts. In terms of differences, in 1990 the breastfeeding rate for non-WIC people was 15.4 percentage points higher than that for WIC clients, while in 2002 that difference rose to 21.1 percentage points. In terms of ratios, the data show that the breastfeeding rate at six months for WIC participants has consistently been only one third to one half the rate for non-WIC participants. The differences, and also the ratios, suggest that on balance WIC participation *retarded *breastfeeding rates for its clients.

The analysis in the Mothers Survey of the Ross Products Division of Abbott is clear:

Nearly half (47%) of all infants born annually in the United States participate in the WIC program, and this number continues to grow. Breastfeeding initiation and duration rates among WIC participants have increased dramatically over the past ten years, but they still lag twenty percentage points behind those of non-WIC participants. This statistic holds true even when controlling for socioeconomic status, geography, race/ethnicity, the age of the mother and birth eight of the baby. In other words, it appears something about the WIC program itself is producing an effect that depresses breastfeeding [[35], p3].

If breastfeeding is best, why is WIC providing formula? WIC's answer is that it is up to their clients to choose. Its position is that it does not encourage formula use, but rather it simply accommodates the wishes of a group that is inclined to use formula. Is this sensible? There are two components to this question. First, is it plausible to believe that WIC is not in fact encouraging formula use? Second, should people who choose an inferior alternative be supported by government in implementing that choice?

Even though WIC is promoting breastfeeding, and does not have any explicit campaign to promote formula feeding, the knowledge that WIC provides free formula is likely to be an important factor in attracting clients to WIC. On that basis, it would not be surprising that WIC clients are more likely to choose formula than those outside the WIC program.

It may be that breastfeeding rates among WIC clients are low ''because breastfeeding is less common among women with lower incomes and less education, and WIC serves this population'' [[36], p15]. However, this does not mean that WIC must act in a way that further encourages feeding with infant formula. The Centers for Disease Control has said:

Public health programs should continue to promote breastfeeding initiation and increase support of breastfeeding continuation, especially among subgroups with the lowest rates (i.e.,black, poor and young mothers; mothers with less than a high school education; and mothers residing in rural areas [[37], p1].

WIC has a breastfeeding promotion program, but its positive impact is diluted by WIC's infant formula program. It is difficult to see how offering free formula could fail to be an incentive to use formula. The inducement is not simply that something of value is being offered at no cost. Even if it is unspoken, there is the implicit message of endorsement: if a government agency is handing out this product, it must be good.

This issue should be examined not only in terms of incentives faced by the clients but also in terms of incentives faced by WIC staff members. WIC staff people are very dedicated. They want to provide services to as many eligible people as possible. Unfortunately, the federal government's funding for the WIC program often has been short, so their offices have not been able to service as many clients as they would like. However, the rebate money from formula has gone a long way toward closing this funding gap. Rebate money now covers the cost of services to about one out of four WIC clients. WIC seemed pleased that in 2001, ''the WIC Program realized over US$1.4 billion in savings generated by infant formula rebates, which allowed over 2.0 million additional participants to be served with the WIC grant'' [[29], p33]. It appears that WIC was highly motivated to get these rebates and thus extend WIC's reach.

This motivation may help to explain why ''in the mid-1990s, several States began awarding their contracts to the bidder offer the highest total rebate'' rather than to the bidder offering the lowest net costs. This provided an incentive to manufacturers to push up their wholesale prices. As a result, a law enacted in November 1997 required that, except under special conditions, the contracts must be awarded to the bidder offering the lowest net price [[29], p33].

WIC staff members have an incentive to encourage the use of formula. Doing so increases the budget they have available to do the work they want to do. This is likely to tip the staff in favor of encouraging clients to select the formula option. The companies are not giving free formula to mothers directly; instead, they are doing that through the WIC program by providing incentives to the program itself.

WIC encourages breastfeeding as the best source of infant nutrition, and it earmarks funds for breastfeeding promotion and support activities. However, the budget for breastfeeding promotion is far less than the amount spent on obtaining formula.

The Food and Nutrition Service contracted with Abt Associates Inc. for a breastfeeding intervention design study to increase breastfeeding among women participating in WIC [38]. The study recommended peer counseling. Apparently the study did not consider the option of ending the distribution of free infant formula as a method for increasing the breastfeeding rate among WIC clients.

This option was also omitted from a study done by the US government's General Accountability Office of the impacts of various methods of marketing of infant formula on breastfeeding rates, especially for WIC clients [39]. Marketing can be understood as the promotion of the use of particular products or services. Surely, supplying products at no cost can be seen as a particularly vigorous form of marketing. Thus, it is not clear why this study of the impacts of various forms of formula marketing failed to assess the impact of providing infant formula at no cost. Also, in its conclusion the study expressed concern about marketing efforts that make improper use of the WIC acronym and logo. The use of the WIC acronym and logo suggests that both formula manufacturing companies and consumers view WIC as endorsing the use of infant formula.

One observer, noting that WIC's breastfeeding promotion efforts have been effective, said, ''the results beg the question of how the rates of breastfeeding initiation and duration might have been different if formula were not being dispensed from the same agency providing the education and support'' [[1], p100]. Overall, the evidence indicates that, whether intended or not, the WIC program has the effect of encouraging formula use.

### International norms

There has been a steady stream of guidance from the international level on the responsibilities of national governments in relation to infant feeding. For example, the *Innocenti Declaration on the Protection, Promotion and Support of Breastfeeding*, adopted in 1990, stated a variety of specific global goals, including the goal that "all women should be enabled to practice exclusive breastfeeding and all infants should be fed exclusively on breast milk from birth to 4–6 months of age'' [[40], p1]. In 1991 the UNICEF Executive Board passed a resolution (1991/22) saying that the Innocenti Declaration would serve as the "basis for UNICEF policies and actions in support of infant and young child feeding.'' In May 1996 the World Health Assembly passed a resolution on Infant and Young Child Nutrition (WHA49.15) in which it confirmed its support for the Innocenti Declaration. The declaration was reaffirmed and updated in 2005 [42].

In 2003, the World Health Organization adopted a *Global Strategy for Infant and Young Child Feeding*. It said, ''As a global public health recommendation, infants should be exclusively breastfed for the first six months of life to achieve optimal growth, development and health. Thereafter, to meet their evolving nutritional requirements, infants should receive nutritionally adequate and safe complementary foods while breastfeeding continues for up to two years of age or beyond'' [[17], p7–8].

In the following sections we focus on two major global sets of norms relating to infant feeding: the *International Code of Marketing of Breast-milk Substitut*es and the human right to adequate food.

### The marketing code

In the 1970s there was a great deal of concern worldwide about the inappropriate marketing and promotion of breast-milk substitutes such as commercial infant formula. There was evidence that formula led to widespread illness and death, especially in poor countries. One major response was adoption of the *International Code of Marketing of Breast-milk Substitutes *by the World Health Assembly on May 21, 1981. The Code should be interpreted in conjunction with a series of subsequent resolutions of the World Health Assembly that help to clarify and extend it [25, 27, 42, 43, 44].

The Code may be summarized as follows:

The International Code of Marketing of Breast-milk Substitutes applies to infant formula and other products marketed or represented as replacements for breast milk, as well as feeding-bottles and teats. The Code prohibits promotion of breast-milk substitutes to the general public, and direct or indirect contact between marketing personnel and pregnant women or mothers of infants and young children. It sets standards for pictures and information on labels, information and educational material on infant feeding, provision of samples and free supplies, and interaction between companies and the health care system [[25], p70].

The Executive Board of the United Nation's Children's Fund (UNICEF) endorsed the Code a few months after its adoption in 1981.

Some formula companies have argued that the Code applies only to poorer countries, but it was intended from the outset to apply to all countries. Some of the strongest affiliates of the Code's strong advocate, the International Baby Food Action Network (IBFAN) are in richer countries, such as Baby Milk Action in Britain and INFACT in Canada [46]. The Code is an explicit part of the infant formula safety standards in several developed countries, such as Britain. Sami Shubber, the World Health Organization's Senior Legal Officer responsible for Code matters at the time of its drafting, was clear on this point:

The International Code was adopted for the whole world and for all the membership of WHO, and there was no question of any distinction between developed and developing countries [44, p44].

Although the US voted against the adoption of the Code in 1981, it has been recognized in some corners of the US government. For example, in 2000 the Department of Health and Human Services put out a *Blueprint for Action on Breastfeeding *as the basis for organizing its breastfeeding promotion activities. It acknowledged:

The marketing of infant formula negatively affects breastfeeding ... The International Code of Marketing of Breast-milk Substitutes and a subsequent WHO resolution delineates guidelines for formula marketing to ensure that it does not interfere with the establishment of lactation. The International Code stipulates the responsibilities of manufacturing industries regarding their role in promoting breastfeeding and appropriate infant feeding practices [45], p17].

Article 11.1 of the Code makes it clear that governments have an important role to play: ''Governments should take action to give effect to the principles and aim of this Code, as appropriate to their social and legislative framework, including the adoption of national legislation, regulations or other suitable measures.''

The Code sees national governments as using the Code in order to control the marketing behavior of commercial enterprises. It does not envision that governments themselves might become significantly involved in the distribution of infant formula, or that governments might do so in inappropriate ways. This might have been anticipated. During the drafting of the *International Code of Marketing of Breast-milk Substitutes*, it appeared that ''The infant food manufacturer's most powerful ally was the US government'' [[25], p62].

While the Code does not explicitly address the possibility of governmental misbehavior with regard to the distribution of breast-milk substitutes, such misbehavior is possible. The premise adopted here is that, at a moral level, the norms that apply to commercial enterprises should generally apply to governments as well.

Many observers have been dismayed at the US government's decision to not support the Code, both domestically and internationally. Typically this is viewed as a matter of omission, a failure on the part of the US government to do some positive things it might have done. However, through its massive distribution of free infant formula, the US government may in fact be the world's largest violator of the principles of the *International Code of Marketing of Breast-milk Substitutes*.

Consider some of the norms set out in the Code.

Article 5 says:

5.1. There should be no advertising or other from of promotion to the general public of products within the scope of this Code.

The Code speaks only about advertising to the general public, and does not say anything about government documents intended for more specialized audiences. Nevertheless, the principle stated in the Code may lead one to question why there are pictures of major brands of formula in a USDA magazine article about the WIC program [23].

Article 6 says:

6.2 No facility of a health care system should be used for the purpose of promoting infant formula or other products within the scope of this Code.

WIC can be viewed as a kind of health care system. On that basis, WIC should not be used to promote the use of infant formula. Giving out free formula certainly must be viewed as a form of promotion.

Article 6 also says:

6.6 Donations or low-price sales to institutions or organisations of supplies of infant formula or other products within the scope of this Code, whether for use in the institutions or for distribution outside them, may be made. Such supplies should only be used or distributed for infants who have to be fed on breast-milk substitutes. If these supplies are distributed for use outside the institutions, this should be done only by the institutions or organisations concerned. Such donations or low-price sales should not be used by manufacturers or distributors as a sales inducement.

World Health Assembly Resolution 47.5 of 1994, which the US government accepted, clarified this when it urged member states ...

... to ensure that there are no donations of free or subsidized supplies of breast-milk substitutes and other products covered by the International Code of Marketing of Breast-milk Substitutes in any part of the health care system [[43], p133].

Since the WIC program can reasonably be viewed as part of the health care system in the US, this means that it should not be accepting free or subsidized supplies of formula.

Article 6.6 says formula should be supplied for free or highly subsidized only for infants "who have to be fed on breast-milk substitutes." Similarly, article 1 says breast-milk substitutes should be used "when these are necessary." "It is only when their use is 'necessary' that they can be properly used for infant feeding" [[43], p57]. WIC distribution is not limited to those infants who *must *be fed on breast-milk substitutes.

Article 6.6 says low-price sales should not be used as a sales inducement. However, it appears that the manufacturers provide formula to WIC at low cost to induce WIC mothers to purchase their brands after they leave the WIC program.

Further, article 6 says:

6.7 Where donated supplies of infant formula or other products within the scope of this Code are distributed outside an institution, the institution or organisation should take steps to ensure that supplies can be continued as long as the infants concerned need them. Donors, as well as institutions or organisations concerned, should bear in mind this responsibility.

WIC provides formula for only a limited period. Women who have left the program must purchase formula on the commercial market.

Article 7 says:

7.3 No financial or material inducements to promote products within the scope of this Code should be offered by manufacturers or distributors to health workers or members of their families, nor should these be accepted by health workers or members of their families.

The rebates constitute a great inducement to WIC staff to accept and possibly encourage the use of infant formula.

Thus, there are several points at which WIC violates the Code. Apart from these specifics, it is important to appreciate that the overall purpose of the Code is to limit inappropriate promotion of the use of infant formula. The massive distribution of infant formula at no cost to mothers that have no special reason to use formula clearly contradicts the spirit and purpose of the Code.

US resistance to the *International Code of Marketing of Breast-milk Substitutes *has been framed primarily in terms of the defense of the free market, and allowing consumers to make their own free choices. The distribution of more than half the formula used in the US through a governmental program at no cost to the consumers is not the result of operation of the free market.

The Code does not call for the prohibition of the marketing of breast-milk substitutes. Instead, it calls for assurance that consumers can indeed make a free and fairly informed choice by getting complete information about the relative merits of feeding their infants through breastfeeding or with breast-milk substitutes. The provision of free formula in massive quantities by a program of the federal government conveys a very persuasive message. The drafters of the Code did not anticipate the form of this message and its source. The distribution of massive quantities of free formula certainly violates the spirit of the Code.

### Human rights

For normative guidance, we can also draw on international human rights law. The most directly applicable international human rights agreement is the *Convention on the Rights of the Child*, an international human rights agreement that came into force in 1990 [46]. Article 24 says, "States Parties recognize the right of the child to the highest attainable standard of health" and they shall take appropriate measures "to combat disease and malnutrition ... through the provision of adequate nutritious foods, clean drinking water, and health care." It also says States Parties shall take appropriate measures "To ensure that all segments of society, in particular parents and children, are informed, have access to education and are supported in the use of basic knowledge of child health and nutrition [and] the advantages of breastfeeding ..." Article 24, paragraph 2a, says that States Parties shall "take appropriate measures to diminish infant and child mortality."

In the *International Covenant on Civil and Political Rights*, which came into force in 1976 [47], article 6 says, "Every human being has the inherent right to life". This clearly implies the right to adequate food and other necessities for sustaining life. Article 11 says that "The States Parties to the present Covenant recognize the right of everyone to an adequate standard of living for himself and his family, including adequate food, clothing, and housing ..." and also recognizes "the fundamental right of everyone to be free from hunger ..."

The fullest articulation of the meaning of the human right to adequate food is in the UN Committee on Economic, Social and Cultural Rights' *General Comment 12 *on *The Right to Adequate Food *[48]. It constitutes a definitive contribution to international jurisprudence. As such, it can be used here as a basis for critically assessing WIC's activities relating to infant formula.

Paragraph 6 provides the basic definition:

6. The right to adequate food is realized when every man, woman and child, alone or in community with others, has physical and economic access at all times to adequate food or means for its procurement. The *right to adequate food *shall therefore not be interpreted in a narrow or restrictive sense which equates it with a minimum package of calories, proteins and other specific nutrients.

Paragraphs 8 and 9 specify some of the dimensions of "adequacy":

8. The Committee considers that the core content of the right to adequate food implies:

The availability of food in a quantity and quality sufficient to satisfy the dietary needs of individuals, free from adverse substances, and acceptable within a given culture;

The accessibility of such food in ways that are sustainable and that do not interfere with the enjoyment of other human rights.

9. *Dietary needs *implies that the diet as a whole contains a mix of nutrients for physical and mental growth, development and maintenance, and physical activity that are in compliance with human physiological needs at all stages throughout the life cycle and according to gender and occupation. Measures may therefore need to be taken to maintain, adapt or strengthen dietary diversity and appropriate consumption and feeding patterns, including breast-feeding, while ensuring that changes in availability and access to food supply as a minimum do not negatively affect dietary composition and intake.

As argued earlier, the consistent evidence of inferior health outcomes with the use of infant formula, when compared with breastfeeding, raises serious doubts about the adequacy of infant formula for meeting the full range of infants' dietary needs.

Paragraph 10 refers to the need to avoid contamination:

10. *Free from adverse substances *sets requirements for food safety and for a range of protective measures by both public and private means to prevent contamination of foodstuffs through adulteration and/or through bad environmental hygiene or inappropriate handling at different stages throughout the food chain; care must also be taken to identify and avoid or destroy naturally occurring toxins.

Infant formula is not packaged as a sterile product. Numerous instances of contamination demonstrate its vulnerability to contamination by adverse substances [49].

Paragraph 11 is concerned with non-nutrient based values:

11. *Cultural or consumer acceptability *implies the need also to take into account, as far as possible, perceived non nutrient-based values attached to food and food consumption and informed consumer concerns regarding the nature of accessible food supplies.

Breastfeeding is not simply about transporting breast milk. The process of breastfeeding, based on sustained bodily contact between mother and infant, is of great psychological value to both infants and their mothers.

Paragraphs 12 and 13 speak about availability and accessibility:

12. *Availability *refers to the possibilities either for feeding oneself directly from productive land or other natural resources, or for well functioning distribution, processing and market systems that can move food from the site of production to where it is needed in accordance with demand.

13. *Accessibility *encompasses both economic and physical accessibility:

Economic accessibility implies that personal or household financial costs associated with the acquisition of food for an adequate diet should be at a level such that the attainment and satisfaction of other basic needs are not threatened or compromised. Economic accessibility applies to any acquisition pattern or entitlement through which people procure their food and is a measure of the extent to which it is satisfactory for the enjoyment of the right to adequate food. Socially vulnerable groups such as landless persons and other particularly impoverished segments of the population may need attention through special programmes.

The WIC program's supply of free formula builds dependency on formula, but provides no help to its clients after they leave the program. For many of them, paying for formula would be a major problem. Thus, receiving free formula for a period of time could make them worse off economically. It would make them worse off not only because of the cost of formula but also because formula feeding is likely to lead to greater health care costs and possibly to lost work time due to illness of the infant.

Paragraph 27 speaks about the obligations of government to regulate businesses:

27. As part of their obligations to protect people's resource base for food, States Parties should take appropriate steps to ensure that activities of the private business sector and civil society are in conformity with the right to food.

Rather than facilitating businesses in inducing families to use feeding methods that are inferior for their infants, governments should be controlling and limiting the ways in which businesses promote infant formula. The *International Code of Marketing of Breast-milk Substitutes *provides clear guidelines for how that should be done.

The *International Code of Marketing of Breast-milk Substitutes *was concluded in 1981, well before the *Convention on the Rights of the Child *came into force in 1990. The Code was not explicitly structured as a human rights agreement. Nevertheless, the Committee on the Rights of the Child, which is the UN treaty body responsible for overseeing the implementation of the convention, working closely with UNICEF, has indicated that it regards the Code as part of the modern human rights framework. The committee uses the Code as one of its guidelines in assessing the performance of countries under the convention.

Apart from the human right to adequate food and the constraints on distribution of formula called for in the Code, there is also a clear human right to life. This is articulated at several points in the international human rights agreements. For example, in the *Convention on the Rights of the Child*, article 24, paragraph 2a calls on States Parties to take appropriate measures "to diminish infant and child mortality." The evidence clearly indicates that in the general population using infant formula tends to increase child mortality. With the government distributing more than half the infant formula used in the US, there is no doubt that government policy is violating children's right to life.

### Applicability of the code and human rights law

This study takes the *International Code of Marketing of Breast-milk Substitutes *and human rights regarding food as major normative frameworks that are appropriate for assessing the activities of the US government with regard to infant formula. This may seem strange, since the US was the only country in the world to vote against the adoption of the Code in 1981, and the US has not ratified either the *International Covenant on Economic, Social and Cultural Rights *or the *Convention on the Rights of the Child*.

The failure of the US government to ratify the *Convention on the Rights of the Child *does not mean that children in the US do not have the same rights as children as elsewhere. From the perspective of the international human rights system, they do have those rights. The failure to ratify simply means that the government has not made a formal commitment to assure the realization of those rights. The convention has not been incorporated into US law, and it cannot be formally invoked in legal proceedings in US courts.

The US government has supported some subsequent resolutions relating to the Code, and some of its agencies have mentioned it in a supportive way, but the Code has never been incorporated into US national law. The US has not adopted the Code as a binding commitment. Nevertheless, the position taken here is that because of the wide global consensus on it, the Code remains meaningful, not as binding law, but as an appropriate moral template through which actions of the US government can be critically assessed.

The Code focuses on the behavior of commercial enterprises, rather than governments. Nevertheless, the Code and the human rights agreements stand as clear articulations of norms, affirmed by most nations of the world. They do not function as binding law within the US, but they do serve as widely accepted templates against which the behavior of all nations may be assessed. These widely accepted international agreements articulate norms relating to the distribution of breast-milk substitutes that has been endorsed, in different ways and in different degrees, all over the world. They are used here not as binding law that establishes specific legal obligations but as clear statements of norms regarding moral responsibilities in the distribution of breast-milk substitutes.

## Summary

### Recommendations

In 1993 the US government's General Accounting Office (now called the General Accountability Office) recommended that the government "develop written policies defining the conditions that would contraindicate breastfeeding and determining how and when to communicate this information to all pregnant and breastfeeding participants of the Special Supplemental Nutrition Program for Women, Infants, and Children (WIC)" [[5], p3]. The resulting study spells out the benefits of breastfeeding, and it provides a detailed analysis of the conditions under which breastfeeding might not be advisable, such as cases in which the mother has specific diseases or has taken certain kinds of drugs. The study concludes by reiterating the benefits of breastfeeding, and recognizing that there are rare situations when the mother should be counseled to not breastfeed. It ends by saying that "Breastfeeding should not be withheld from any infant unless absolutely necessary" [[5], p32].

What happened with these recommendations? Surely one must recognize the contradiction between acknowledging that mothers should only rarely be counseled to not breastfeed, and at the same time providing free infant formula to very large numbers of mothers.

Perhaps people should have the opportunity to choose to use infant formula, just as they are allowed to choose greasy hamburgers and cigarettes. The point here is that allowing a questionable product to be on the market is one thing. Having the government promote it is quite another. Having the government promote infant formula particularly among poor people raises enormous ethical questions. Does the balance of benefits and risks from the use of infant formula justify the government's providing infant formula to almost half the infants in the US?

Even if they ask, WIC will not provide alcoholic beverages to its clients. The fact that they might ask for beer, for example, is not a sufficient reason to provide it. Similarly, the fact that some WIC clients prefer to use infant formula is not a sufficient justification for WIC to provide it. The large-scale distribution of free infant formula by WIC to all clients who ask for it is a situation that needs to be fixed.

If infant formula could be demonstrated to produce better infant health, there might be a reason to distribute it without cost to those who could not otherwise afford it. However, there is no evidence to support the generalization that the use of infant formula results in better infant health than breastfeeding. On the contrary, the evidence clearly and consistently shows that the use of infant formula increases the risks of morbidity and mortality throughout the life cycle. The use of infant formula has been shown to be harmful to the health of mothers as well. The inescapable conclusion is that the government should not be distributing free infant formula.

It might be argued that if they were not supplied with infant formula, some WIC clients might instead use juice, cow's milk, evaporated milk, or over-diluted formula. There is that risk, but it is likely to be overcome with proper breastfeeding support, from WIC, employers, and others. Moreover, those who feel that they must use infant formula would remain free to purchase infant formula. It does not seem sensible to promote an inferior product simply because one can imagine something that is even worse.

WIC could reasonably provide infant formula, but only in exceptional cases, if that is recommended for a specific reason by a physician or a lactation counselor. An initial list of acceptable reasons could be drawn from the Lawrence study of contraindications to breastfeeding [5]. The rebate program should be ended. The WIC program should stop providing free infant formula to the majority of its clients, and should be limited to providing infant formula to no more than a small percentage of its clients. WIC should provide the best possible information to all its clients about the relative merits of different ways of feeding their infants, and it should monitor health and other impacts of different feeding methods. Following a well-designed transition plan [[1], p101], WIC could stop providing infant formula to most of its clients.

Some mothers would complain if the prospect of getting free infant formula were cut off, but they would still be free to purchase it on their own in the regular retail market. The companies surely would fight any change in policy that reduces the flow of formula. However, the US government's duty here is to stand up for the infants. Currently, WIC acts in a way that benefits commercial enterprises at the expense of infants. The government should not risk infants' health in order to support commercial enterprises.

## Competing interests

The author(s) declare that they have no competing interests.
